# First Trimester Scan: “Pyramid of Priorities” of the Brazilian Reality

**DOI:** 10.1055/s-0038-1673368

**Published:** 2018-10

**Authors:** Pedro Pires, Edward Araujo Júnior

**Affiliations:** 1 Department of Gynecology and Obstetrics, Universidade de Pernambuco, Recife, PE, Brazil; 2 Department of Obstetrics, Paulista Medicine School, Universidade Federal de São Paulo, São Paulo, SP, Brazil

The assessment of nuchal translucency (NT) and other markers for chromosomal disorders in the first trimester scan has been overvalued in Brazil, while the assessment of the crown-rump length (CRL) and early morphology are not given the same attention. The contribution of these markers to the antenatal care routine is evident. Similarly, the contribution of the inverted “pyramid” of care proposed by Nicolaides^1^ has been valuable in prenatal care, demonstrating the importance of ultrasound parameters in the first trimester of pregnancy.

**Fig. 1:**
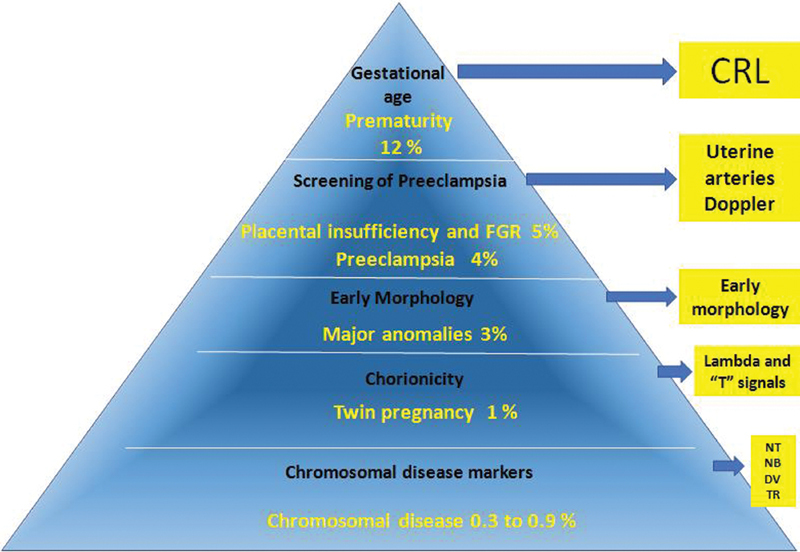
New pyramid of priorities for the first trimester scan in Brazil.

Parallel to the pyramid proposed by Nicolaides,^1^ we propose a new “pyramid of priorities” including ultrasound for morphological findings in the first trimester (Fig. 1).

This new “pyramid” draws attention to the main perinatal outcomes and to the priority of each assessment according to the perinatal impact of each parameter. Prematurity is one of the main perinatal health issues, and the most important cause of neonatal morbidity and mortality. The situations of greater adverse impact on perinatal outcomes, particularly in underdeveloped or developing countries, include prematurity (12%), preeclampsia (7%), placental insufficiency and/or fetal growth restriction (5%), congenital malformations (3%), twin pregnancy (1%), and chromosomal disorders (0.3 to 0.9%).^2^ ^3^ ^4^ ^5^ ^6^ Notably, preeclampsia, placental insufficiency and/or fetal growth restriction, congenital malformations, twin pregnancy, and chromosomal disorders can all lead to prematurity. Preterm birth, placental insufficiency, and fetal anomalies account for 90% of perinatal deaths. In addition to the adverse perinatal outcomes, preeclampsia is the leading cause of maternal mortality in developing countries, and the third cause of maternal mortality in the United States of America (20%).^4^ ^6^

Considering that prematurity is associated with placental insufficiency, which in turn is associated with fetal growth restriction, the challenge of preventing and screening for these conditions becomes even greater. The prevalence and perinatal impact of prematurity and fetal growth restriction are much greater than those of chromosomal disorders (< 1%), which, unlike preeclampsia and prematurity, are known to be non-susceptible to prevention and prophylaxis. These obstetric complications are as common today as they were 40 years ago, and this highlights the challenges in the use of appropriate screening methods and the implementation of appropriate strategies to reduce their prevalence.

The assessment of NT and other markers for chromosomal disorders, as encouraged by European centers with different economic and public health situations, is not performed by many sonographers in Brazil. Moreover, while performing the assessment, the risk calculation recommended by the Fetal Medicine Foundation is mostly not conducted.^7^ Furthermore, NT assessment demands a long learning curve. We often receive first trimester scan requests mentioning NT alone, indicating that this measurement may be anticipated as the most important parameter to be evaluated in this period. Thus, drawing the attention of medical societies to the CRL as the most important parameter of the first trimester scan is imperative. In addition to providing precise information on gestational age, with a margin of error of 3 to 5 days, the CRL has a short learning curve, and it is quick, reproducible, and the only parameter with significant impact on perinatal outcomes.^8^ Furthermore, from 10 weeks to 13 weeks and 6 days, ultrasound with CRL measurement for the accurate assessment of gestational age has a level of evidence A.^9^

Undeniably, all obstetric reasoning and conduct depend on the precise estimation of the gestational age to enable safe fetal growth monitoring, adequate obstetric care, management of prematurity, and identification of fetal growth abnormalities.^10^ ^11^ The overvaluation of NT in Brazil indicates that its request and routine assessment are challenging at several public institutions, since many doctors who conduct ultrasound scans have no specific training for this measurement, and many pregnant women miss their opportunity to have the CRL measured to properly assess the gestational age. The importance of assessing uterine artery Doppler in the first trimester should also be emphasized because it far outweighs the impact of measuring NT,^12^ ^13^ given the prevalence of preeclampsia and the possibility of prophylaxis with the use of aspirin, which has been proved to be effective when introduced before 16 weeks of gestation in randomized clinical trials.^14^ ^15^

Therefore, we suggest that public institutions withdraw the request for NT measurement from the first trimester scans. The constrained demand for first trimester scans due to the inclusion of the NT measurement request may thus be reduced. Regarding the ultrasound report, we suggest that the reference to NT is maintained; however, its measurement is optional, and depends on the examiner's skills.
